# Novel Drug Delivery Method Targeting Para-Aortic Lymph Nodes by Retrograde Infusion of Paclitaxel into Pigs’ Thoracic Duct

**DOI:** 10.3390/cancers14153753

**Published:** 2022-08-01

**Authors:** Akira Saito, Natsuka Kimura, Yuji Kaneda, Hideyuki Ohzawa, Hideyo Miyato, Hironori Yamaguchi, Alan Kawarai Lefor, Ryozo Nagai, Naohiro Sata, Joji Kitayama, Kenichi Aizawa

**Affiliations:** 1 Department of Surgery, Jichi Medical University, Tochigi 329-0498, Japan; saito.akira@jichi.ac.jp (A.S.); ykaneda819@jichi.ac.jp (Y.K.); 92015ho@jichi.ac.jp (H.O.); hideyomiyato3810@icloud.com (H.M.); yamaguchi@jichi.ac.jp (H.Y.); alefor@jichi.ac.jp (A.K.L.); sata2018@jichi.ac.jp (N.S.); kitayama@jichi.ac.jp (J.K.); 2 Division of Clinical Pharmacology, Department of Pharmacology, Jichi Medical University, Tochigi 329-0498, Japan; kimura_n@jichi.ac.jp; 3 Division of Translational Research, Clinical Research Center, Jichi Medical University Hospital, Tochigi 329-0498, Japan; 4 Jichi Medical University, Tochigi 329-0498, Japan; rnagai@jichi.ac.jp; 5 Clinical Pharmacology Center, Jichi Medical University Hospital, Tochigi 329-0498, Japan

**Keywords:** thoracic ducts, paclitaxel, thoracic duct infusion

## Abstract

**Simple Summary:**

For advanced cancer, surgery may not be possible at the site of lymph node metastasis, such as para-aortic lymph node metastasis. Systemic administration of anticancer drugs has been performed in these cases, but treatment results are still inadequate. This study investigated the efficiency of drug delivery to intra-abdominal lymph nodes by administering an anticancer drug retrogradely to lymphatic vessels in order to deliver the drug directly to the metastatic lymph nodes. Thoracic duct infusion resulted in the same concentration of paclitaxel in abdominal lymph nodes as via systemic administration, but the serum concentration was lower. The results show that thoracic infusion may achieve higher paclitaxel doses than systemic administration. Infusion of anti-cancer drugs into the thoracic duct may yield clinical benefits for patients with extensive lymphatic metastases in abdominal malignancies.

**Abstract:**

Gastrointestinal cancer with massive nodal metastases is a lethal disease. In this study, using a porcine model, we infused the anti-cancer drug Paclitaxel (PTX) into thoracic ducts to examine the efficiency of drug delivery to intra-abdominal lymph nodes. We established a technical method to catheterize the thoracic duct in the necks of pigs. We then compared the pharmacokinetics of PTX administered intrathoracically with those of systemic (intravenous) infusion. Serum, liver, and spleen concentrations of PTX were significantly lower following thoracic duct (IT) infusion than after intravenous (IV) administration approximately 1–8 h post-infusion. However, PTX levels in abdominal lymph nodes were maintained at relatively high levels up to 24 h after IT infusion compared to after IV infusion. Concentrations of PTX in urine were much higher after IT administration than after IV administration. After IT infusion, the same concentration of PTX was obtained in abdominal lymph nodes, but the serum concentration was lower than after systemic infusion. Therefore, IT infusion may be able to achieve higher PTX doses than IV infusion. IT delivery of anti-cancer drugs into the thoracic duct may yield clinical benefits for patients with extensive lymphatic metastases in abdominal malignancies.

## 1. Introduction

Lymphatic metastasis usually occurs in the direction of the lymph flow. In abdominal malignancies, such as gastrointestinal or ovarian cancer, metastatic tumor cells in regional lymph nodes move to the retroperitoneal para-aortic nodes and then spread to the left neck lymph nodes (Virchow’s nodes) through the thoracic duct [1,2]. Extensive lymph node metastases (ELM), including para-aortic lymph node metastases, are commonly regarded as unresectable. These malignancies are treated with a combination of surgery, radiotherapy, and systemic chemotherapy. However, outcomes of patients with ELM are still very poor in gastric [3,4,5], pancreatic [6], and colorectal [7,8] cancer. In comparison, prognoses of patients with ELM from ovarian cancers tend to be better, which is likely due to their greater chemosensitivity [9,10]. It is possible that exposure of metastatic lymph nodes to higher concentrations of anti-cancer drugs could improve outcomes of patients with ELM, even for gastrointestinal cancers.

Recently, new routes of administration have been developed for the administration of anticancer drugs. The intraperitoneal administration of Paclitaxel (PTX) has been performed for peritoneal dissemination in gastric cancer cases, producing excellent clinical results. [11,12] Therefore, we considered the retrograde administration of PTX from lymphatic vessels as a novel route of administration for ELM in the abdomen. PTX is a broad-spectrum anticancer drug that is clinically effective against lung, liver, ovarian, breast, and other cancers. However, its solubility is less than 0.1 μg/mL in water, which seriously affects its bioavailability. [13] PTX injection uses castor oil as a solvent for enhancing its solubility; however, castor oil inevitably induces hypersensitivity and toxicity. [14,15] PTX for injection (albumin-bound) has a low drug load (10%), although it has a low toxicity and good tolerance. Thus, improving its water solubility and bioavailability with effective preparation and delivery is a worthy scientific endeavor.

The thoracic duct, the body’s central lymphatic vessel, originates in the cisterna chyli in the retroperitoneum, ascends between the esophagus and the descending aorta in the mediastinum, and flows into the left venous angle in humans [16,17]. We hypothesized that high doses of anti-cancer drugs might be administered with low systemic toxicity if given via the thoracic duct in retrograde fashion, so as to selectively infuse metastatic lymph nodes in the retroperitoneum. Based on this hypothesis, we catheterized the thoracic ducts of pig necks and infused PTX via catheters. We then compared the pharmacokinetics of PTX administered intrathoracically with those of systemic (intravenous) infusion.

## 2. Materials and Methods

### 2.1. Drugs and Animals

PTX (Taxol) was purchased from Bristol-Myers Squibb Japan (Tokyo, Japan). The PTX used is a commercial product, with 100 mg of PTX dissolved in 8.35 mL of polyoxyethylene castor oil and 8.35 mL of ethanol. Porcine models, comprising 4 female pigs with an average body weight of 28.2 kg (range: 20.8–36.0 kg), were purchased from Sanesu Breeding Co., Ltd. (Funabashi, Japan) and were housed individually at the Center for Development of Advanced Medical Technology (CDAMTech), Jichi Medical University. All animal handling procedures in this study complied with the Jichi Medical University Guide for Laboratory Animals, the Guide for the Care and Use of Laboratory Animals published by the U.S. National Institutes of Health (NIH Publication, eighth edition, 2011), and the ARRIVE guidelines [18]. The Institutional Animal Care and Concern Committee at Jichi Medical University approved all experimental protocols (Approval number: 20139-01).

### 2.2. Porcine Thoracic duct Cannulation Model

**Figure 1:**
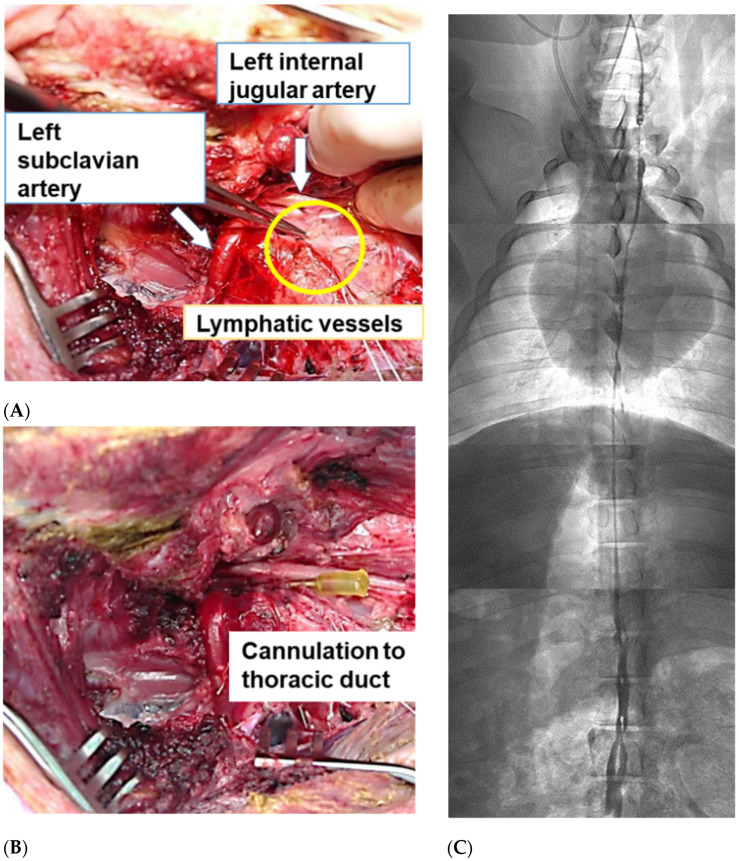
Thoracic duct cannulation. The lymphatic vessel was identified between the left internal carotid artery and left subclavian artery at their bifurcation (**A**), and the terminal thoracic duct was cannulated with a 24G needle (**B**). The whole length of the thoracic duct was visualized by injecting the radiocontrast, Omnipaque (**C**). An image comprising four separate images of the neck, chest, thoracoabdominal junction, and abdomen intraoperatively using a fluoroscopic system (Mobile C-arms). The four images were stitched together to form a composite image.

After the pigs were placed in a supine position under inhalation anesthesia with sevoflurane, a 15 cm longitudinal incision was made 1 cm to the left of the midline at the 1/3 level on the cranial crest, caudal side. The left anterior cervical muscle was dissected, the left external jugular vein and the left subclavian vein were ligated and dissected, and then the first rib was dissected and removed 2 cm from the sternum attachment. We then performed a laparotomy, and 2 mL of Patent Blue (FUJIFILM Wako Pure Chemical Corporation, Osaka, Japan) were injected into the mesenteric lymph nodes of the small intestine using a fine needle. Cervical lymphatic vessels could be identified in the remaining left internal carotid artery and left subclavian artery bifurcation a few minutes after the dye injection. Discharge of the lymph was confirmed after cannulation with a 24G SURFLO needle was performed (Figure 1A,B). The thoracic duct was identified by the C-arm due to injection of the radiocontrast OMNIPARK (Figure 1C). PTX (dose: 30 mg) was dissolved in physiological saline (50 mL) and was administered at a flow rate of 100 mL/h using an automatic infusion pump (NIPRO, Sakai, Japan).

### 2.3. Measurement of PTX Concentrations in Various Organs with High-Performance Liquid Chromatography–Mass Spectrometry (LC-MS/MS)

After intravenous administration of 100 mg of hydrocorton (Nichiiko, Tokyo, Japan), 30 mg of PTX (MOCHIDA, Tokyo, Japan) were administrated from the cannulated thoracic duct, as described above. Pigs were systematically cannulated in the left internal jugular vein with a 24G Surflo needle, into which PTX was then intravenously infused. At 1, 3, 8, 12, and 24 h post-infusion, blood and urine samples were collected and centrifuged at 2,000 rpm for 10 min at 4 °C. Supernatants were then collected, placed in cryotubes, and stored at −80 °C. After thawing the samples, PTX concentrations were measured with high-performance liquid chromatography–mass spectrometry (LC-MS/MS).

Fifty µL of each liquid sample (serum, lymph, urine), to which 200 µL of methanol and 20 µL of internal standard reagent (PTX-d5, 1 µg/mL-MeOH) had been added, were vortexed for 30 s and then centrifuged (12,000 rpm for 10 min at 4 °C). Supernatants were then subjected to spin filtering. Filtrates were analyzed by LC-MS/MS (LCMS-8050 System, Shimadzu, Kyoto, Japan). Tissue samples (10 mg), to which 20 µL of internal standard reagent (PTX-d5, 1 µg/mL-MeOH) and 1 mL of 0.1% formic acid–MeOH had been added, were homogenized and centrifuged (12,000 rpm for 10 min at 4 °C). Supernatants (500 µL) were transferred to other tubes, to which 200 µL of water had been added. Samples were then vortexed and centrifuged (12,000 rpm for 10 min at 4 °C), and supernatants were analyzed.

For LC analyses, a YMC-Triart C_18_ analytical column (50 × 2 mm, 1.9 µm) was used, and the column oven and autosampler were set to 40 °C and 4 °C, respectively. Mobile phase A was 0.1% formic acid-water, and mobile phase B was acetonitrile. The flow rate was set to 0.5 mL/min, and the injection volume was 3 μL. The gradient was 40% B in 2.7 min, ramping up to 100% by 4 min, remaining at 100% until 6 min, and then decreasing over 0.5 min, followed by an equilibration step at 40% B for 1.5 min.

PTX and PTX-d5 were detected in ESI positive mode. MS/MS conditions were as follows: Nebulizer gas flow (3 L/min), heating gas flow (10 L/min), interface temperature (300 °C), desolvation temperature (526 °C), heat block temperature (400 °C), and drying gas flow (10 L/min). Collision energies were 24 V, 22 V, and 21 V for PTX, and 24 V, 25 V, and 21 V for PTX-d5. PTX, and PTX-d5 were observed at m/z 854 > 286 and m/z 859 > 291, respectively. PTX concentrations of the biological samples were quantified by the area ratio with the internal standard reagent (PTX-d5) added to the samples.

**Figure 2:**
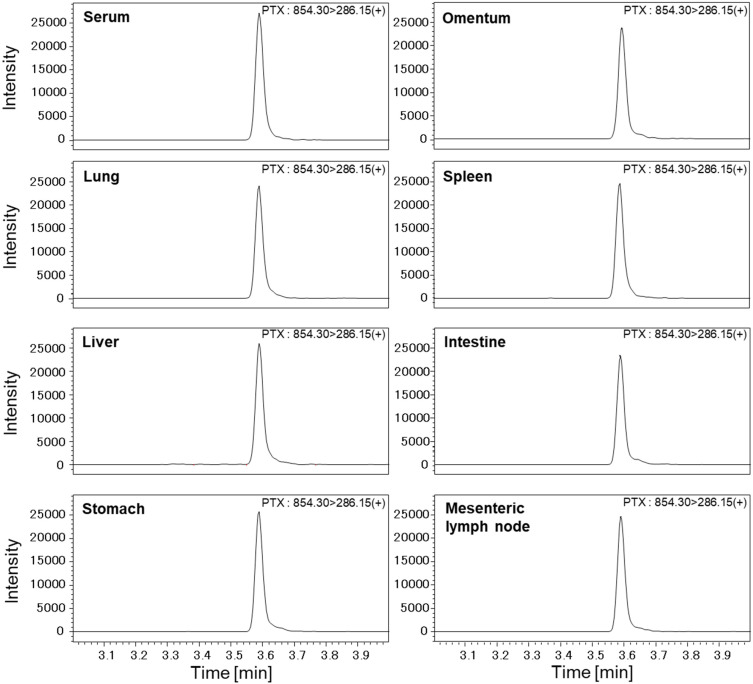
Representative LC-MS/MS chromatograms of transition m/z 854.30 to 286.15 on positive ion mode (+) for PTX. The peak at 3.59 min shows the response of the signature peptide transition. PTX (0.01 µg/mL) was added to porcine serum and tissue extract, and the recovery test was evaluated by adding PTX at the concentration.

PTX was added to the serum at a concentration of 0.005–5 µg/mL to prepare an 8-point calibration curve. The accuracy and linearity of the calibration curve, carryover, diurnal variation, inter-day variation, and sample stability were confirmed. Recovery tests of PTX added to porcine serum (4 animals) were performed, and accuracies were 110% for porcine serum (Figure 2). For urine analyses, PTX was added to pig urine, derived from pigs to which PTX had not been administered, at concentrations of 0.125–1 µg/mL to prepare an 8-point calibration curve.

Concentrations in various organs were measured in the early and late phases, since frequent tissue collection with such an invasive procedure could seriously affect pharmacokinetics. In late phase analysis, some organs were taken at 8, 12, and 24 h after PTX infusion. Para-aortic lymph nodes were additionally taken only at the end of the experiment. Immediately after collection, each organ was stored in its collected state in a cryotube at −80 °C. Later, 10 mg samples of these organs were homogenized, and PTX concentrations were measured, as described above.

### 2.4. Statistical Analysis

Results were analyzed using Student’s *t*-tests, and *p* values < 0.05 were considered statistically significant.

## 3. Results

### 3.1. Concentrations of PTX in Serum and Urine

**Figure 3:**
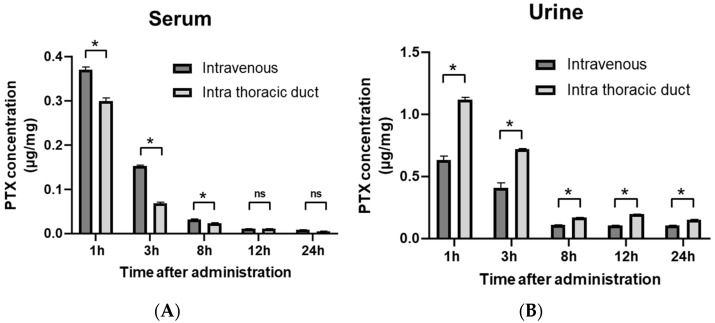
PTX concentrations in serum (**A**) and urine (**B**) after IT or IV injection. Serum and urine samples were obtained 1 to 24 h after infusion. PTX concentrations were measured with a mass spectrometer (LC-MS/MS). Data show the mean ± SD of 3 experiments. *: *p* < 0.01, ns: not significant.

Immediately after PTX was administrated through the intrathoracic duct (IT), considerable amounts of PTX were detected in sera. However, concentrations of PTX 1 to 12 h after administration were significantly lower than those after intravenous administration (IV). The PTX concentration decreased with time, ultimately disappearing from the serum after 24 h with both IT and IV administration (Figure 3A). In contrast, concentrations of PTX in urine samples were significantly higher with IT administration than with IV administration at all times (Figure 3B).

### 3.2. PTX Concentrations in Various Organs

**Figure 4:**
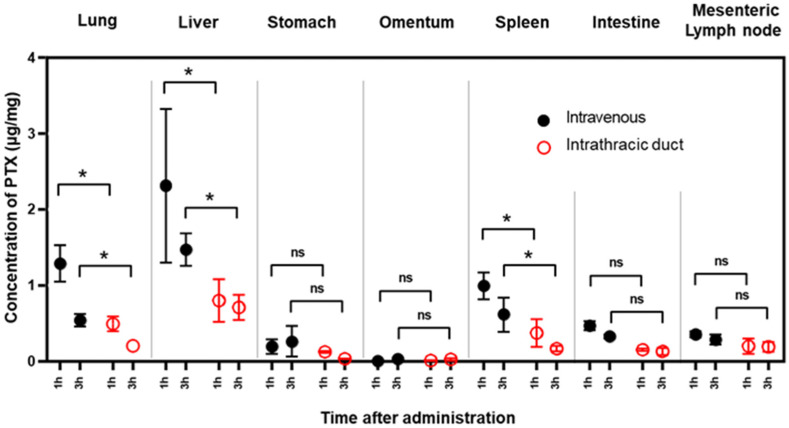
Concentrations of PTX in various organs soon after IT or IV injection. Three samples were obtained from each organ 1 and 3 h after infusion, and PTX concentrations were measured with a mass spectrometer (LC-MS/MS). Data show the mean ± SD of 3 samples. *: *p* < 0.01, ns: not significant.

In the first set of experiments, PTX accumulation in various organs was examined at 1 and 3 h post-infusion. In the lungs, liver, and spleen, PTX concentrations after IV administration were more than twice as high as after IT administration. However, there were no significant differences between the two infusion methods in terms of PTX concentrations in mesenteric lymph nodes (Figure 4).

**Figure 5:**
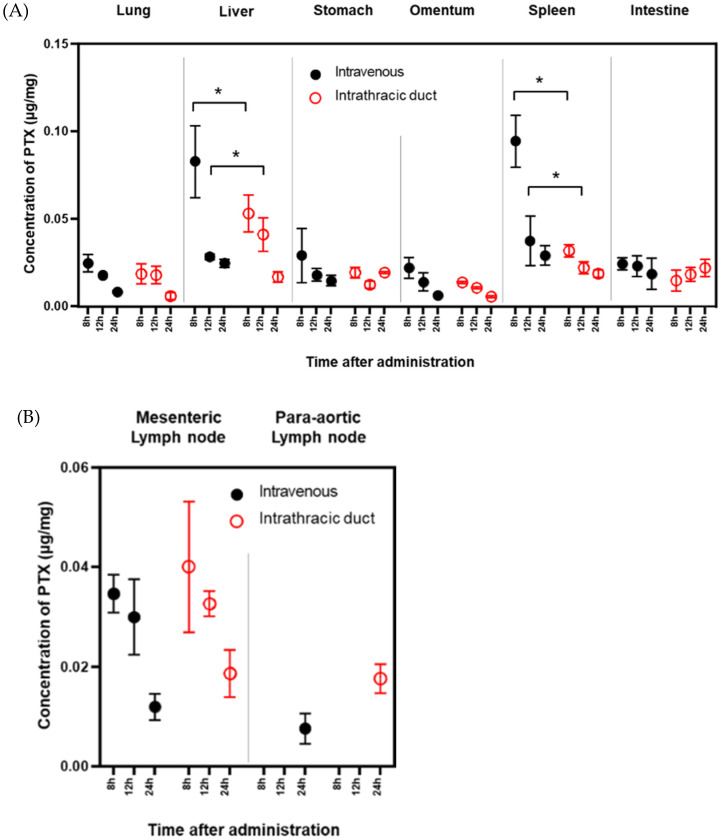
PTX concentrations in various organs (**A**) and various lymph nodes (**B**) at later time points after IT or IV injection. Three samples were obtained from each organ 8, 12, and 24 h after infusion. PTX concentrations were measured with a mass spectrometer (LC-MS/MS). Data show the mean ± SD of three samples. *: *p* < 0.01.

In the next set of experiments, we compared PTX concentrations 8, 12, and 24 h after IV and IT infusion. Although PTX concentrations were still high in the liver and spleen at 8 h, there were no differences in PTX levels in any organs between IV and IT administration after 12 h. However, 24 h after IT administration, relatively high PTX concentrations were still present in mesenteric and para-aortic lymph nodes compared to those following IV administration (Figure 5A,B).

## 4. Discussion

The thoracic duct is the main collecting vessel of the lymphatic system, and it drains lymph from the abdomen and lower extremities into the venous blood stream [19]. Thus, the thoracic duct is a direct pathway to the retroperitoneal lymph nodes through the cisterna chyli. In fact, clinical studies have documented retrograde spreading of esophageal and lung cancers to abdominal lymph nodes through the thoracic duct [20,21]. These findings inspired us to examine the possibility of retrograde intrathoracic duct chemotherapy for gastrointestinal cancers with extensive metastases to the retroperitoneal lymph nodes.

We succeeded in cannulating the terminal thoracic duct in pig necks, and attempted to achieve the selective retrograde administration of PTX to the lymphatic system. We selected PTX because the concentration of PTX in the lymph in the thoracic duct is maintained at high levels after intraperitoneal infusion, probably due to its high molecular weight and hydrophobic properties [22]. Using this minimally invasive model, we were able to compare pharmacokinetics in various organs between intrathoracic (IT) and intravenous (IV) infusions of drugs up to 24 h after infusion. The pigs were divided into an IV group and an IT group, and three samples were obtained from these animals. The pigs were sacrificed immediately after the experiment. We observed no apparent morbidity/complications for at least 24 h after the start of surgery. Our hypothesis was that the IT administration of PTX would result in much higher PTX accumulation in abdominal lymph nodes than IV administration, with less loss to circulating blood. In fact, within 12 h of infusion, PTX concentrations in the serum, liver, and spleen were significantly lower with IT than with IV administration; however, this difference was smaller than expected. Comparing the two routes, no significant differences were detected in PTX concentrations in abdominal lymph nodes, the stomach, intestines, or omentum. Moreover, concentrations of PTX in mesenteric or para-aortic lymph nodes tended to be higher after IT than IV administration, 24 h after infusion. This suggests that higher doses of PTX can be given via IT routes without systemic toxicity, as compared with IV treatment, which is pharmacokinetically advantageous. Based on these results, the IT administration of PTX appears to be a useful method to deliver PTX to metastatic lymph nodes located in the abdominal cavity and retroperitoneum.

On the other hand, PTX concentrations in urine were considerably higher with IT than IV administration, especially at early time points. This suggests that most IT-infused PTX was retrogradely transported to the renal cortex and excreted from the kidneys, since the renal lymphatic outflow is connected to the para-aortic lymph nodes through the thoracic duct [23]. However, urine volume (2160 mL) with IT during surgery was decreased compared with urine volume (4890 mL) in IV administration. This result suggests that IT administration increased thoracic duct pressure, decreased atrial natriuretic peptide [24], and increased urinary PTX concentration.

In summary, we established an excellent experimental model to assess the pharmacodynamics of IT-administrated drugs. Using this system, we examined the drug delivery efficiency of the IT infusion of PTX for extensive metastatic lymph nodes in the abdomen. After IT infusion, the same concentrations of PTX were obtained in abdominal lymph nodes, but with lower serum concentrations than after IV infusion. Thus, it may be possible to achieve higher PTX doses with IT than with IV administration. However, the amount of urinary excretion may present a risk of renal toxicity and requires further study. When anti-cancer drugs suitable for IT administration are found, this method may be ideal for the treatment of patients with extensive para-aortic lymph node metastases without distant metastases to other organs.

## 5. Conclusions

Using a novel pig model, we found that the IT administration of anti-cancer drugs may offer a significant pharmacokinetic advantage and has clinical merit for patients with extensive lymph node metastases (ELM), including para-aortic lymph node metastases from abdominal malignancies.

## Data Availability

The data presented in this study are available on request from the corresponding author.
